# Novel Self-Healing Superhydrophobic Coating with Oil–Water Separation and Anti-Icing Properties

**DOI:** 10.3390/nano14241981

**Published:** 2024-12-10

**Authors:** Xiuge Wang, Lulu Tang, Shumin Fan, Wenxiu Fan

**Affiliations:** 1 School of Biological Engineering, Xinxiang Institute of Engineering, Xinxiang 453700, China; wxg78@126.com; 2 School of Chemistry and Chemical Engineering, Henan Institute of Science and Technology, Xinxiang 453003, China; 21202201147@stu.hist.edu.cn (L.T.); fansm88@hist.edu.cn (S.F.)

**Keywords:** self-healing, superhydrophobic, durable, Fe_3_O_4_ nanoparticles

## Abstract

A self-healing superhydrophobic coating was successfully prepared in the present work. The coating comprised PEG (polyethylene glycol) and Fe_3_O_4_ nanoparticles modified with stearic acid (SA) via hydrogen bonds, using polyamide resin and epoxy as binders. The chemically damaged surface could restore its original superhydrophobic structure and chemical composition after 4 h at room temperature or 10 min of heating in an oven with a self-healing efficiency of 95.5% and 96.1%, respectively. The hydrogen bonds between SA-OH and Fe_3_O_4_-OH nanoparticles enabled the repeatable and efficient self-healing properties of the superhydrophobic coating. The coating exhibited remarkable chemical resistance, maintaining superhydrophobicity even after 48 h of immersion in strong acidic and alkaline solutions. Additionally, the prepared fabric showed excellent mechanical stability after 2400 mm of abrasion and 125 cycles of tape peeling with a WCA above 150°. Furthermore, the coated fabric was effective for oil/water separation and anti-icing. With these powerful functions, the proposed superhydrophobic coating holds promising applications in both daily life and industry.

## 1. Introduction

Nature-inspired superhydrophobicity refers to surfaces with water contact angles (WCAs) greater than 150° and sliding angles (SAs) lower than 10° [1,2]. Superhydrophobic surfaces have garnered considerable attention for their significant potential in oil/water separation [3], self-cleaning [4], anti-biofouling [5], and anti-icing [6]. It has been reported that superhydrophobic coatings can be fabricated by creating hierarchical roughness and modifying the surface with low-energy reagents [7]. Various fabrication methods, including electrospinning [8], etching [9], spraying [10], electrodeposition [11], chemical vapor deposition [12], and hydrothermal treatment [13], have been explored. However, these coatings often suffer from structural fragility under harsh conditions, such as exposure to light and corrosive liquids [14]. Therefore, there is an urgent need to develop facile approaches to prepare robust and highly stable superhydrophobic coatings [15].

To enhance the durability of superhydrophobic coatings, researchers have explored the use of thermosetting polymers to improve mechanical stability and adhesion [16]. Others have focused on incorporating self-healing functions into superhydrophobic materials, which has proven more efficient [17]. External stimuli, such as light [18], heat [19], pH changes [20], solvents [21], applied voltage [22], or high humidity [23], are often required to trigger the self-healing capability of coatings formed by covalent networks, such as fluoroalkylsilane (FAS). Qiu et al. [14] developed a self-healing superhydrophobic surface composed of polydimethylsiloxane (PDMS), Polyfluo-150Wax, and palygorskite, which healed scratches under high-temperature treatment. Zhao et al. [24] fabricated a self-repairing superhydrophobic coating with 1H, 1H, 2H, 2H-perfluorodecanethiol (PFDT) modified SiO_2_ using a spraying approach, where the wettability could be restored at 80 °C after surface damage. Zhang et al. [25] created a self-healing superhydrophobic coating on an aluminum substrate using spraying and hydrothermal reaction methods. The chemical damage healing process required 40 min at room temperature. The migration of hydrophobic chains in the low-surface-energy material FAS@PDA provided self-repairing capability, which could be enhanced by heating. Sun et al. [26] designed a self-healing superhydrophobic coating using silica particles modified with polycaprolactone, epoxy resin, EASTMAN Kristalex 3085 resin, and 1H, 1H, 2H, 2H-perfluorodecyltriethoxysilane (PFDTES) via a spray-coating method, significantly extending the lifespan of the coatings. However, commonly used self-healing agents, such as fluorinated compounds, can be harmful to humans and the environment and are costly. Additionally, achieving rapid self-repairing ability for superhydrophobic coatings without external stimuli remains a challenge.

Hydrophobic bonds, being dynamic and reversible non-covalent bonds, are easily broken and reformed due to their lower energy compared to covalent bonds [27,28]. Hydrogen bonds can form when there are abundant O-H groups present [29]. Self-healing superhydrophobic coatings based on hydrogen bonds can restore both chemical and physical damage [30]. In this study, Fe_3_O_4_ nanoparticles were modified with PEG and stearic acid (SA) via hydrogen bonds. The PEG/Fe_3_O_4_-SA nanoparticles were applied to cotton fabric using epoxy and polyamide resins. Following further modification with SA, a superhydrophobic coating was successfully prepared. Although the superhydrophobic properties could be lost after 48 h of immersion in corrosive solutions, they could be restored by simple heat treatment or storage at room temperature. The mechanical stability of the prepared coating was investigated, and its application in reusable oil/water separation was demonstrated. This method provides a simple route to design durable self-healing superhydrophobic coatings with great potential for industrial applications.

## 2. Materials and Methods

### 2.1. Materials

Stearic acid (SA), tetrachloromethane (CCl_4_), polyethylene glycol (PEG, Mn 6000), Fe_3_O_4_ nanoparticles, hydrochloric acid (HCl), potassium bromide (KBr), sodium hydroxide (NaOH), and anhydrous ethanol (AE) were purchased from Shanghai Macklin Biochemical Co., Ltd. LC, Shanghai, China. Fabric, cotton, sponge, copper, and aluminum were obtained from the local market. Deionized water was prepared in the laboratory.

### 2.2. Preparation of Superhydrophobic Fabric

**Figure 1:**
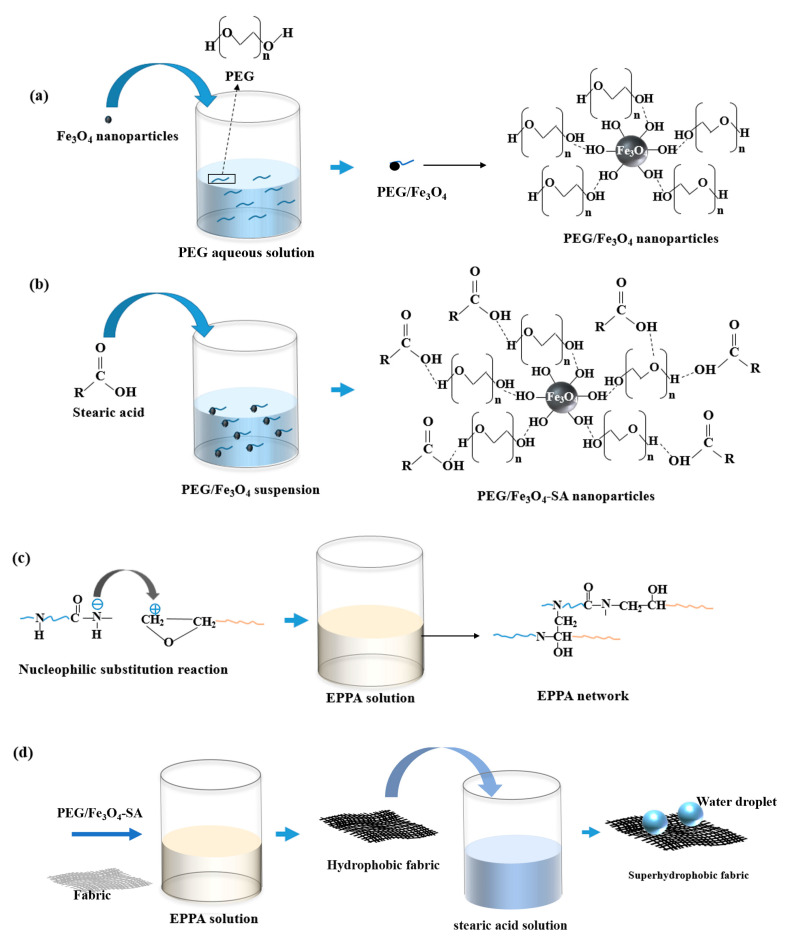
Schematic diagram of the fabrication process for superhydrophobic fabric: (**a**) synthesis of PEG/Fe_3_O_4_ nanoparticles; (**b**) synthesis of PEG/Fe_3_O_4_-SA nanoparticles; (**c**) EPPA binder formation; (**d**) superhydrophobic coating on fabric.

The fabrication process is illustrated in Figure 1. A 5 × 5 cm fabric sample was pretreated by washing with anhydrous ethanol and deionized water, and then dried in an oven. The first step involved preparing PEG/Fe_3_O_4_ nanoparticles. An amount of 1.5 g of PEG was dissolved in 100 mL of deionized water to obtain PEG aqueous solution. The Fe_3_O_4_ nanoparticles were immersed in PEG aqueous solution for 2 h and dried at 80 °C in an oven for 3 h to obtain PEG/Fe_3_O_4_ nanoparticles. Then, the prepared PEG/Fe_3_O_4_ nanoparticles were dissolved in anhydrous ethanol. An amount of 0.6 g of SA was added to the solution, which was then stirred at 70 °C for 2.5 h. The resulting precipitates were dried at 80 °C in an oven, yielding superhydrophobic Fe_3_O_4_ nanoparticles (PEG/Fe_3_O_4_-SA). In the second step, 3 g of epoxy and 2 g of polyamide resins were mixed in 80 mL of anhydrous ethanol solution, followed by sonication and stirring for 2 h to obtain an EPPA solution through a nucleophilic substitution reaction. The third step involved preparing the superhydrophobic fabric. The PEG/Fe_3_O_4_-SA was added to the EPPA solution, followed by sonication for 1 h. The fabric was then immersed in the solution for 2 min and dried at room temperature for 2 h. Finally, the dried fabric was immersed in an ethanol solution of stearic acid (3.6 wt.%) for 1 h, yielding the superhydrophobic fabric after drying at room temperature. The fabrication process of the superhydrophobic coating on other substrates was identical to that of the superhydrophobic fabric.

### 2.3. Oil/Water Separation

The superhydrophobic fabric proved to be an excellent candidate for oil/water separation. The prepared fabric was fixed at the top of a beaker for separating and collecting oil. An amount of 10 g of ethyl acetate colored with Sudan III was used as the oil, which was mixed with water in the separation process, simulating a floating spill. The separation efficiency (η) was calculated using the following equation:

$$\eta =\frac{M_{2}-M}{M_{1}}\times 100\%$$

where *M* is the sum weight of the beaker and superhydrophobic fabric; *M*_1_ is the weight of the oil before separation; and *M*_2_ is the sum weight of separated oil, the beaker, and superhydrophobic fabric.

### 2.4. Characterization

The morphology of the samples was examined using scanning electron microscopy (SEM, Quanta 200, FEI, Hillsboro, OR, USA). The chemical structure of the samples was analyzed using Fourier-transform infrared spectroscopy (FTIR, Magna-IR 560, Thermo Nicolet, Madison, WI, USA) and X-ray photoelectron spectroscopy (XPS, Escalab 250Xi, ULVCA-PHI, Chigasaki, Kanagawa, Japan). The surface wettability was evaluated using a water contact angle measuring instrument (TST-200H, Shenzhen testing equipment CO., Ltd., Shenzhen, China). The WCAs were measured by using 5 μL of deionized water droplets on the sample surface, which were determined by averaging 3 values from different positions of each sample.

## 3. Results and Discussion

### 3.1. Surface Morphology of Samples

**Figure 2:**
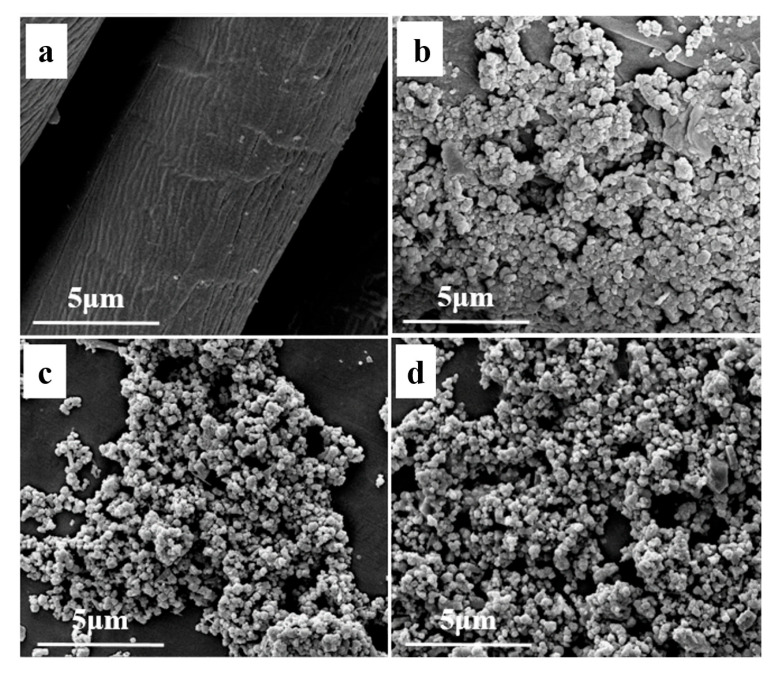
SEM images of (**a**) pristine fabric, (**b**) superhydrophobic fabric, (**c**) Fe_3_O_4_ nanoparticles, and (**d**) PEG/Fe_3_O_4_-SA nanoparticles.

Surface morphology is crucial for fabricating wettability. Thus, analyzing the surface morphology of superhydrophobic materials is essential. Figure 2 shows the surface topography of pristine fabric, superhydrophobic fabric, Fe_3_O_4_ nanoparticles, and PEG/Fe_3_O_4_-SA nanoparticles. Figure 2a reveals a clear and smooth surface for the pristine fabric. In contrast, a rough structure with micro-protrusions appears on the surface of the superhydrophobic fabric (Figure 2b). The pristine Fe_3_O_4_ nanoparticles (Figure 2c) range from 0.1 to 0.2 μm. The surface morphology of the PEG/Fe_3_O_4_-SA nanoparticles (Figure 2d) remains as a layer of fine nanoparticles. With the assistance of EPPA binders, PEG/Fe_3_O_4_-SA nanoparticles are modified on the fabric surface, forming a porous microstructure after ethanol evaporation.

**Figure 3:**
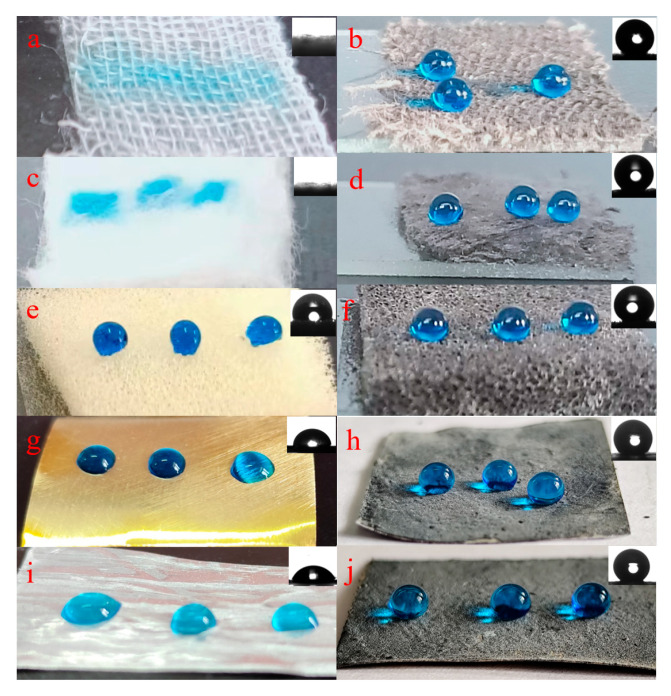
The pristine fabric (**a**), cotton (**c**), sponge (**e**), copper (**g**), and aluminum (**i**) and corresponding superhydrophobic samples (**b**,**d**,**f**,**h**,**j**).

The superhydrophobic coating was applied to different substrates besides fabric, including cotton, sponge, copper, and aluminum. The superhydrophobic copper and aluminum were prepared by the spraying method. The superhydrophobic samples based on a variety of substrates are shown in Figure 3. The WCAs of superhydrophobic pristine fabric, cotton, sponge, copper, and aluminum were 0°, 0°, 113.7°, 70.3°, and 84.9°, respectively. However, the WCAs of superhydrophobic samples were 154.8°, 153.2°, 161.2°, 151.1°, and 151.6°, proving great application potential of the superhydrophobic coating for varieties of substrates. The fabric with an EPPA solution followed by immersion in a stearic acid solution was used as a blank. The WCA of the blank fabric sample was 127.7°, proving the contribution of the PEG/Fe_3_O_4_-SA nanoparticles to superhydrophobic properties.

**Figure 4:**
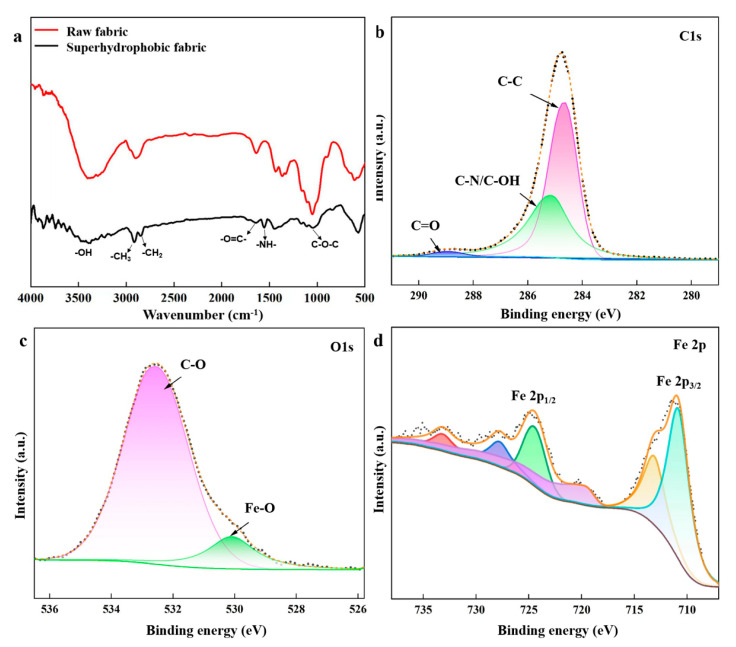
(**a**) FTIR spectra of samples. XPS spectra of superhydrophobic: (**b**) C1s; (**c**) O1s; (**d**) Fe2p. (The peaks in XPS analysis were fitted, and the color and lines were to make characteristic peaks of elements more visible).

Figure 4a shows the FTIR spectra of the samples. In the spectra of raw and superhydrophobic fabric, the peak appearing between 3200 and 3500 cm^−1^ corresponds to the stretching vibrations of the –OH group in fabric. The superhydrophobic fabric exhibits new peaks compared to raw fabric. For the superhydrophobic fabric, 2960 cm^−1^ and 2870 cm^−1^ correspond to the symmetrical and asymmetrical stretching vibrations of –CH_3_ and –CH_2_ groups in stearic acid. The peak at 1000–1100 cm^−1^ corresponds to the stretching vibration of the C-O-C group, which is the characteristic absorption peak of PEG. Peaks at 1510–1570 cm^−1^ and 1640–1690 cm^−1^ correspond to the –N–H and O=C– bending vibrations of the O=C–NH– group in the EPPA binder. Based on this analysis, stearic acid, EPPA, and PEG are characterized and confirmed, demonstrating the successful grafting of these agents onto the fabric surface.

The chemical nature of the superhydrophobic fabric was investigated using XPS. The spectra in Figure 4b–d depict the presence of C, O, and Fe elements in the superhydrophobic fabric. In Figure 3b, the C1s spectra are decomposed into four peaks: C-C (284.5 eV), C-O (286.4 eV), C=O (288.2 eV), and C-N/C-OH (285.5 eV). The C-C, C-O, and C=O bonds are related to PEG, EPPA, and SA, while the C-N bond originates from EPPA. From the Fe 2p orbitals in Figure 3d, peaks at 714.3 and 727.3 eV are attributed to Fe^3+^, and those at 711 and 724.4 eV are attributed to Fe^2+^, confirming the existence of Fe_3_O_4_.

### 3.2. Self-Healing Performaces

**Figure 5:**
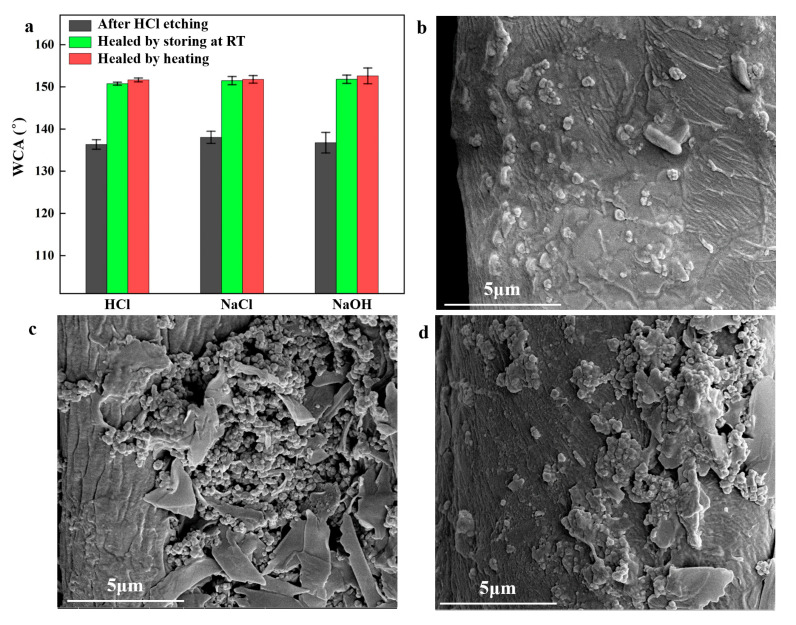
(**a**) Recovery of WCA etched by HCl, NaCl, and NaOH; SEM images of the coating after immersion in 0.1 M HCl (**b**) and 0.1 M NaOH (**c**) and 0.1 M NaCl (**d**) for 48 h.

The coating’s self-healing capability was evaluated by immersing it in strong acid (0.1 M HCl), alkali (0.1 M NaOH), and saline (0.1 M NaCl) solutions for 48 h. Variations in WCAs of etched and self-healed coatings are shown in Figure 5a. When the fabric was immersed in 0.1 M HCl solution for 48 h, the WCA of the etched coating dropped to 136.4°. After storing the etched fabric at room temperature for 4 h, the WCA stabilized at 150.7°. Heating the etched fabric in an oven at 80 °C for 10 min increased the WCA to 151.6°. The self-healing efficiency, calculated as the ratio of the WCA of the original to the healed fabric, was 95.5% and 96.1% for room temperature and oven heating, respectively. Similar self-healing efficiencies were obtained for coatings etched by NaCl and NaOH solutions. After immersion in NaCl and NaOH solutions, WCAs decreased to 138.0° and 136.8°, respectively. However, heating increased the WCAs of the healing surfaces to 151.8° and 152.6°. SEM images of surfaces etched by immersion in 0.1 M HCl, 0.1 M NaOH, and 0.1 M NaCl for 48 h are shown in Figure 5b–d. Although some micro/nano-bulges were lost during immersion, the micro/nanostructure was largely retained. Therefore, the superhydrophobic coating demonstrated excellent anti-corrosion and self-healing capabilities.

**Figure 6:**
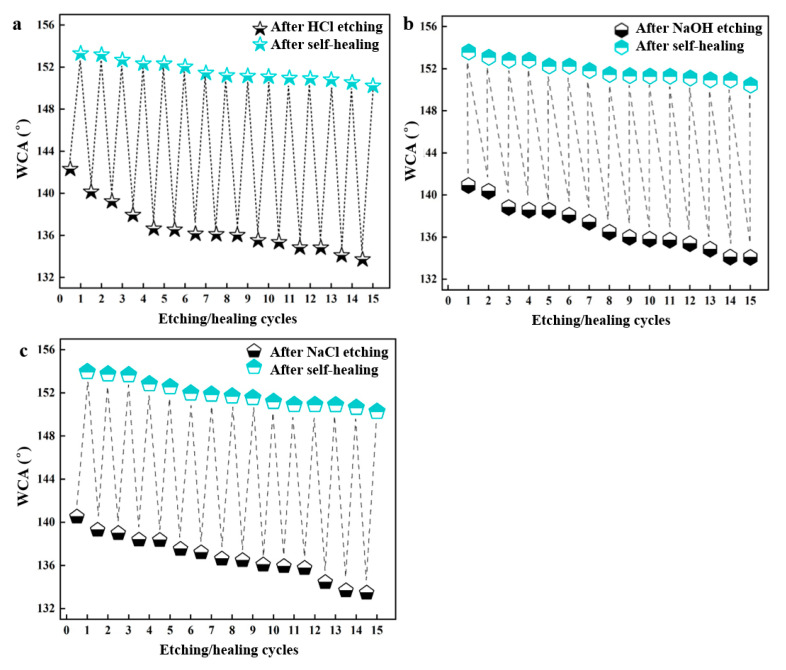
Variations in WCA in etching–healing cycles: (**a**) HCl; (**b**) NaOH; (**c**) NaCl.

The stability of the superhydrophobic coating was further characterized by repeating the etching/self-healing processes (Figure 6). After immersion in 0.1 M HCl, 0.1 M NaOH, and 0.1 M NaCl for 4 h, the WCAs of the coating were measured, followed by heating for recovery. The initial superhydrophobic coating switched to a hydrophobic state after immersion but returned to its superhydrophobic state after heating. The etching/self-healing processes were conducted for 15 cycles with no significant decrease in superhydrophobicity, demonstrating excellent cycling stability.

**Figure 7:**
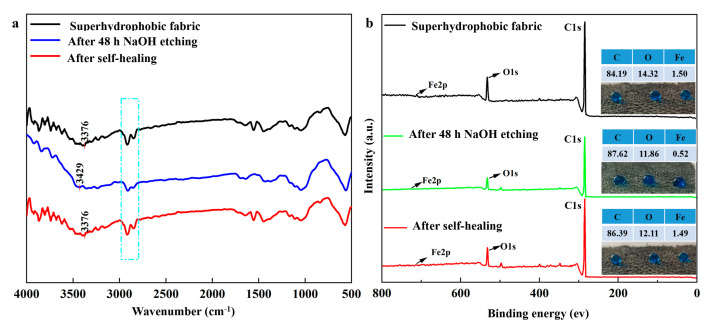

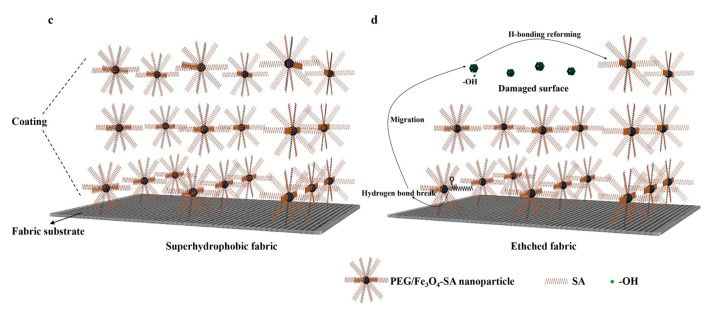
(**a**) FTIR and (**b**) XPS spectra of raw, etched, and healed coatings; (**c**) schematic diagram of superhydrophobic coating structure; (**d**) schematic diagram of proposed mechanism of self-healing process.

To explain the self-healing mechanism of the prepared superhydrophobic coating, FTIR and XPS spectra of superhydrophobic fabric, etched fabric after 48 h of immersion in 0.1 M NaOH solution, and healed fabric after heating treatment were analyzed. Figure 7a shows the FTIR analysis, comparing the chemical composition variations before and after self-healing. The C-H peaks of the etched fabric decreased compared to the superhydrophobic fabric. The O-H peaks of the etched fabric blue-shifted from 3376 cm^−1^ to 3429 cm^−1^, indicating the loss of hydrogen bonds between Fe_3_O_4_-OH nanoparticles and SA-OH. After healing, the C-H peaks increased, and the O-H peaks red-shifted from 3429 cm^−1^ to 3376 cm^−1^, proving the out-migration of SA and its reintegration with Fe_3_O_4_-OH groups through hydrogen bonds. XPS spectra (Figure 7b) showed that the C, O, and Fe contents of the superhydrophobic fabric were 84.19%, 14.32%, and 1.50%, respectively. For the etched fabric, these contents changed to 87.62%, 11.86%, and 0.52%. After healing, the contents were 86.39%, 12.11%, and 1.49%, respectively, similar to those of the superhydrophobic fabric. Water droplets on the etched fabric flattened with a WCA of 133.5°, whereas those on the healed fabric increased to 152.0°, restoring superhydrophobic properties.

A possible self-healing mechanism is illustrated in Figure 7c,d. Figure 7c shows the pristine structure of the superhydrophobic coating. After immersion in a corrosive solution, the outer layer was damaged (Figure 7d), partially losing SA from the coating, and exposing −OH groups. The damage of hydrogen bonds and removal of SA increased surface free energy. To minimize surface free energy, SA migrated to the outer damaged surface, reforming hydrogen bonds between SA-OH and PEG/Fe_3_O_4_ nanoparticles, thereby recovering superhydrophobic properties through the rearrangement of low-surface-energy chains in SA. A significant number of SA molecules connected through hydrogen bonds in the micro/nanostructure is crucial for the coating’s self-healing properties.

### 3.3. Mechanical Durability

**Figure 8:**
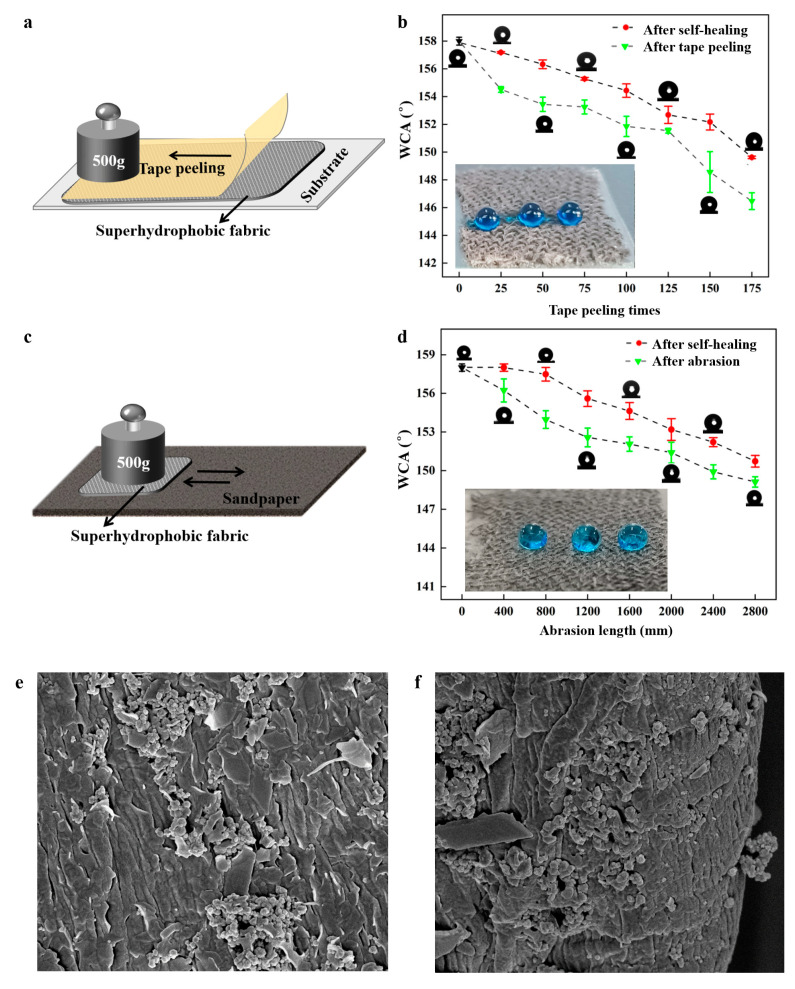
(**a**) The tape peeling process; (**b**) WCAs as a function of tape peeling times; (**c**) abrasion process; (**d**) WCAs as a function of abrasion length; (**e**) SEM image of the coating after 175 tape peeling cycles; (**f**) SEM image of the coating after 2800 mm of abrasion. The insets are the water droplets on fabrics after 175 tape peeling cycles and 2800 mm of abrasion.

Mechanical durability is usually a challenge for superhydrophobic materials in practical applications. In this study, two durability tests were conducted to assess the mechanical robustness and self-healing properties of the fabricated coating. The tape peeling test was applied to test the degradation of the superhydrophobic surface (Figure 8a). A 500 g weight was rolled over the tape surface repeatedly, and the WCAs of the coated surface were measured every 25 cycles, followed by heating for self-healing. As shown in Figure 8b, the coating maintained excellent superhydrophobicity (WCA > 151.6°) even after 125 cycles of tape peeling. The WCA decreased to 148.4° after 150 cycles but increased to 152.3° after self-healing, indicating exceptional mechanical resistance. Additionally, a sandpaper abrasion test was conducted to evaluate the coating’s abrasion resistance (Figure 8c). A 500 g weight was applied to the superhydrophobic coating against 1000-grit sandpaper and moved back and forth for 100 mm. Figure 7d shows that the WCAs of the superhydrophobic coating remained above 150° after 2400 mm of abrasion. After 2800 mm of abrasion, the coating lost superhydrophobicity. After self-healing, the WCA recovered to 151.2°. These results demonstrated excellent mechanical abrasion resistance. Tang et al. [31] used methyltrimethoxysilane (MTMS) to prepare superhydrophobic wood by the immersion-spraying method. The WCA decreased to 150° after 1200 mm of sandpaper abrasion. Hou et al. [32] studied the mechanical durability of the superhydrophobic PVDF- HFP/SiO_2_/CNTs coating on the Al substrate. The tape peeling cycles were repeated 30 times. The WCA decreased from 165.7° to 155.1°. Zhao et al. [33] fabricated a robust superhydrophobic anti-icing/de-icing composite coating. The surface lost its superhydrophobic properties with a WCA below 150° after 2500 mm of sandpaper abrasion. The surfaces prepared in this study maintained excellent superhydrophobic properties and their rough structure after 175 tape peeling cycles or 2800 mm of abrasion (Figure 8e,f), illustrating strong adhesion between the coating and fabric.

### 3.4. Oil/Water Separation

**Figure 9:**
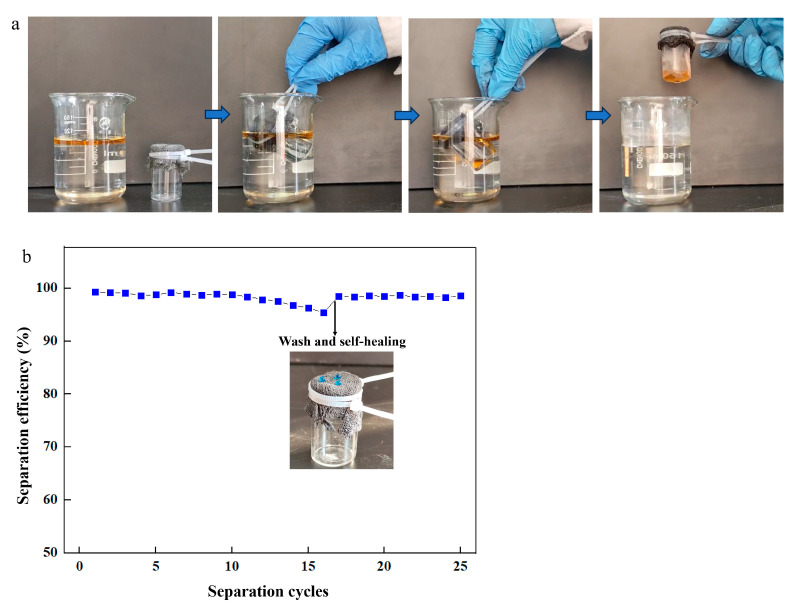
(**a**) Oil/water separation process; (**b**) separation efficiency with separation cycles.

To evaluate the application of the prepared coating, the superhydrophobic fabric was used for oil/water separation (Figure 9a). Oil penetrated the superhydrophobic fabric into the beaker, retaining water outside during separation. The process was repeated to assess performance. After 10 cycles, the separation efficiency remained nearly unchanged at 98.7%, proving stable performance (Figure 9b). The separation efficiency decreased to 95% after 16 cycles. The used superhydrophobic fabric was washed with AE and heated at 80 °C for 10 min for self-healing. The healed fabric was reused for repeated separation processes, maintaining high efficiency even after 25 cycles, indicating its significant development potential and wide application in oil/water separation.

### 3.5. The Anti-Icing Ability of the Coating

**Figure 10:**
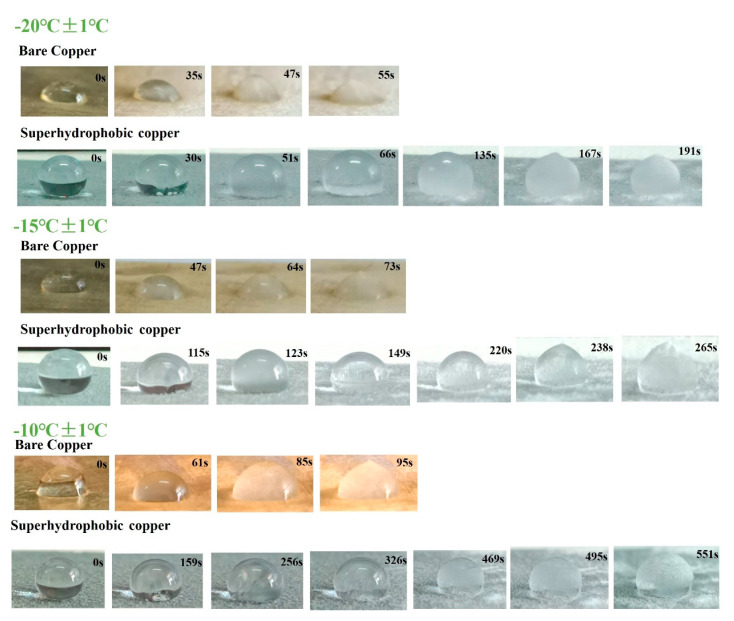
The freezing process of water droplets on the surface of bare copper and superhydrophobic copper.

Water droplets on bare copper and superhydrophobic copper were observed to freeze in the low-temperature condition. The time of ice forming was monitored. Figure 10 shows the photos of the freezing process of a 25 μL water droplet on different surfaces. In the freezing process, water droplets changed from transparent to hemispherical and to a peach shape. For bare copper, the droplet showed a peach shape at 73 s at a temperature of −15 °C, illustrating the total freezing of the droplet. The water droplets on superhydrophobic copper showed a peach shape at 238 s at a temperature of −15 °C, exhibiting a longer delay during the freezing process. Superhydrophobic properties could enhance the removal of droplets and minimize the contact area on surfaces, as well as reduce nucleation sites. This ultimately contributes to a delay in freezing time.

## 4. Conclusions

A self-healing superhydrophobic coating was successfully prepared. The fabric was coated with SA-Fe_3_O_4_ nanoparticles by immersion, followed by further modification with SA, achieving remarkable superhydrophobic properties with a WCA of 154.8°. The coating demonstrated excellent chemical resistance even after 48 h of corrosion in strong acidic, alkaline, and saline solutions. The self-healing performance was attributed to the breakage and reintegration of hydrogen bonds between SA-OH and Fe_3_O_4_-OH nanoparticles. Only 10 min of heating was needed for the self-healing process, restoring the damaged surface to the original superhydrophobic structure. Moreover, the superhydrophobic fabric exhibited outstanding mechanical durability even after 175 tape peeling cycles or 2800 mm of abrasion. The self-healing superhydrophobic fabric proved to be a good candidate for oil/water separation and anti-icing. Consequently, a stable, durable, and efficient self-healing superhydrophobic coating was designed, significantly enhancing reliability for real-life applications and extending lifespan.

## Data Availability

The data presented in this study are available on request from the corresponding author.
